# Functionalized magnetic particles for water treatment

**DOI:** 10.1016/j.heliyon.2019.e02325

**Published:** 2019-08-21

**Authors:** Christian Baresel, Vincent Schaller, Christian Jonasson, Christer Johansson, Romain Bordes, Vinay Chauhan, Abhilash Sugunan, Jens Sommertune, Sebastian Welling

**Affiliations:** a IVL Swedish Environmental Research Institute AB, Box 210 60, Stockholm, 100 31, Sweden; b RISE Acreo, Arvid Hedvalls Backe 4, Gothenburg, Sweden; c Department of Chemistry and Chemical Engineering, Chalmers University of Technology, Gothenburg, Sweden; d RISE Surface, Process and Formulation, Box 5607, Stockholm, SE-114 86, Sweden

**Keywords:** Environmental science, Chemical engineering, Nanotechnology, Materials chemistry, Magnetic particle, Water treatment, Life cycle assessment, Pollutant

## Abstract

In this study, we have taken the concept of water treatment by functionalized magnetic particles one step forward by integrating the technology into a complete proof of concept, which included the preparation of surface modified beads, their use as highly selective absorbents for heavy metals ions (Zinc, Nickel), and their performance in terms of magnetic separation. The separation characteristics were studied both through experiments and by simulations. The data gathered from these experimental works enabled the elaboration of various scenarios for Life Cycle Analysis (LCA). The LCA showed that the environmental impact of the system is highly dependent on the recovery rate of the magnetic particles. The absolute impact on climate change varied significantly among the scenarios studied and the recovery rates. The results support the hypothesis that chelation specificity, magnetic separation and bead recovery should be optimized to specific targets and applications.

## 1 Introduction

The global water management situation is alarming and requires urgent and innovative technologies to ensure a proper treatment of raw, process and sewage waters. In the coming decades, problems with accessing good quality water are expected to worsen. Water scarcity is predicted to occur globally, even in regions currently considered water-rich. Addressing these problems has triggered a tremendous amount of research to identify robust methods of purifying water at lower cost and with less energy, while at the same time minimizing the total impact on the environment.

A growing number of contaminants are entering water supplies as a result of human activity. This includes traditional compounds such as heavy metals and emerging micropollutants such as pharmaceutical residues and endocrine disruptors. Heavy metals have been excessively released into the environment due to rapid industrialization and have become a major issue due to their persistence in the environment and toxicity to many life forms. It constitutes an immense problem to which a proper solution has not yet been found (Cleuvers, 2003; Isidori et al., 2006; Kloepfer et al., 2005; Metcalfe et al., 2001; Triebskorn et al., 2007; Zhang et al., 2008). Heavy metals tend to accumulate in living tissues and do not degrade into harmless products, which causes various diseases and disorders (Ayangbenro and Babalola, 2017; Dixit et al., 2015; Gaur et al., 2014; Tak et al., 2013). A variety of industries, such as metal processing, finishing and plating, produce wastewater that often contains heavy metals of particular concern for human health, including zinc, copper, nickel, mercury, cadmium, lead and chromium. To protect human health and the environment, regulations often require removing heavy metals from various industrial wastewaters before discharging to the environment.

The current technology for the removal of heavy metals from wastewater is inefficient. Mostly the use of polyelectrolytes that enable chemical precipitation, membrane filtration, and adsorption techniques are applied. These processes have significant disadvantages and limitations, including the incomplete removal of heavy metals, high operational cost, high-energy consumption, and a production of toxic sludge. In most of the cases, the recovery of the adsorbent is not possible or not cost effective.

It is in this context that the application of magnetic nanoparticles has appeared as a promising approach about 15 years ago. Combined with proper surface modification, the magnetic nanoparticles offer the possibility of a facile separation after capturing the pollutants. Developed initially for analytical purposes, for which the facile separation was an asset to quantify pollutant levels (Zhao et al., 2008; Šafařík and Šafaříková, 2002), the concept of magnetic nanoparticles as adsorbents in water treatment processes has become increasingly popular (Li et al., 2013; Tang and Lo, 2013).

Tailor-made magnetic nanoparticles are recognized due to their unique properties and potential for modification (Fonnum et al., 2005; Pankhurst et al., 2003; Pinheiro et al., 2018; Santhosh et al., 2016; Simeonidis et al., 2016). In the scope of water remediation, they have key properties that generally provide opportunities to efficiently target and remove pollutants that current treatment technologies fail to achieve. These are:

⁃ They possess large surface-to-volume ratios, which enhance the efficiency in terms of the quantity used to capture a specific contaminant;
⁃ They may show superparamagnetic properties meaning that the particles get magnetized only in the presence of a field and have no residual magnetization after the field is removed, which prevents the clustering of particles. They also have a high magnetic saturation value and a high magnetic susceptibility that provides a high particle magnetic moment. Contaminant-loaded magnetic particles can therefore be easily separated from the solution via an external magnetic field and gradient;
⁃ Their surface can be modified with inorganic shells and/or organic molecules. Not only does this stabilize the particles and prevent their oxidation, but it also provides specific functionalities such as increased affinity towards target contaminants;
⁃ The possibility to tune the surface chemistry to condition the recyclability of the bead system. This implies enabling the release of the bound contaminant and re-use of the particles in multiple water treatment cycles, hence minimizing the environmental impact of the method;
⁃ Magnetic micro- and nano-meter sized particles based on iron oxides have been established in biomedical and medical applications for over two decades (e.g., for biomagnetic separation, immunoassays, and as contrast agents in medical imaging techniques). Comprehensive in vitro/in vivo toxicity studies have already been carried out. Thus, an extensive database on the toxicity of magnetic particles has been developed which helps to develop a process with no environmentally harmful wastes or byproducts.

Nowadays the most classical approach consists of realizing hybrid materials where a magnetic core will facilitate the separation while the rest of the composite is meant to provide specific adsorption and a high specific surface area, thus capitalizing on the above-mentioned properties (Harikishore Kumar Reddy and Lee, 2014; Lu et al., 2017; Peng et al., 2014; Song et al., 2018).

Despite several studies on the use of magnetic nanoparticles for water depollution, the concept has remained, at best, an appealing proof of concept. No global approach was taken to take into the account the potential of such technology in a real-life scenario. Therefore, our aim has been, through an inter-disciplinary approach, to combine the preparation and the study of the extraction capacity of the surface functionalized magnetic beads based on magnetic nanoparticles, with an investigation of the separation performance. The data gathered was used to develop different scenarios that served as input to a life cycle assessment study, thus allowing the identification of the key parameters in the further development and up-scaling of this technology.

## 2 Materials and methods

Iron oxide nanoparticles, to be incorporated into polymeric bead particles to provide magnetic functionality, were prepared via NaBH_4_ reduction of iron acetylacetonate solution. Details of the process are described elsewhere (Sommertune et al., 2015). Hydrogen evolution during the reaction of NaBH_4_ with water created a reducing environment that prevents Fe(II) oxidation to Fe(III), thereby improving the air-stability of the magnetite nanoparticles during the reaction. The nanoparticles were accumulated with a magnet placed under the reaction container and the remaining solution containing the reaction by-products was drained away. The nanoparticles were then re-dispersed with deionized water. This process was done three times to ensure that all the unwanted reaction byproducts were removed.

The magnetic cores were then incorporated into polymeric (polystyrene) microbeads and the magnetic particles were prepared by an emulsion solvent evaporation (ESE) process. Table 1 summarizes the different magnetic particles prepared.

**Table 1:** Summary of magnetic particles prepared.

	Core (nm)	Bead (nm)	Surface chemistry
MB01	7	1950	Polymeric surfactant
MB02	7	1050	Polymeric surfactant
MB006	25	1300	SDS and amino-acid based surfactant; 1:3
MB007	25	1100	SDS and amino-acid based surfactant; 1:1

The magnetic particles were then functionalized with divalent amino acid-based surfactants. The N-acyl surfactants were prepared by coupling aminomalonic acid, aspartic acid, and glutamic acid, three amino acids bearing two carboxylic groups, and differing solely in the size of the spacer between the carboxylic groups, with a fatty acid moiety, yielding N-acyl amino acid surfactants (Bordes et al., 2009; Bordes and Holmberg, 2015).

The magnetic properties of the particles produced have been studied and compared with commercially available particle systems. The main property addressed is how fast the particles can be separated in a liquid (water) using an external magnetic field. How different magnetic setups affect the separation time has also been investigated both by simulations and by experiments. For this, a lab-scale separation system for testing different magnetic concepts, flow rates and different particles was used. The sample magnetic moment versus applied field over the sample up to 1 T at room temperature for each particle system was measured using a vibrating sample magnetometer (VSM) from Lakeshore Cryotronics. The measured sample magnetic moment was then normalized to the total particle mass to obtain the mass magnetization (mean particle magnetic moment per mass of particle) and to the number of particles to obtain the mean magnetic moment per particle (the result is given in Fig. 3a,b). A light attenuation/transmittance setup (Schaller et al., 2008) was used to measure the concentration of magnetic particles in liquids when they are separated by a permanent magnet. The change in particle concentration in a sample volume could thus be monitored over time by measuring the light transmittance and thereby determine the separation time (the result is given in Fig. 5). The combination of particle separation times and the magnetic properties of the particles are plotted in Fig. 6.

In addition, several FEM-models have been developed and implemented in COMSOL to simulate the magnetic separation process. The models were used to study different separation concepts and to understand the important parameters that have impact on separation efficiency. First, the separation-time measurement setup was modelled to verify the simulation of our approach by comparing it with the experimental results. The results showed that the model has certain limitations and consistently overestimates the separation time. The main reason for that is the complexity of the physical problem that we are trying to model. We have not been able to include all factors affecting the separation process (e.g. particle cooperation effects/particle chain building). However, the models provided a good qualitative understanding and helped in the optimization of the separation system.

**Fig. 1:**
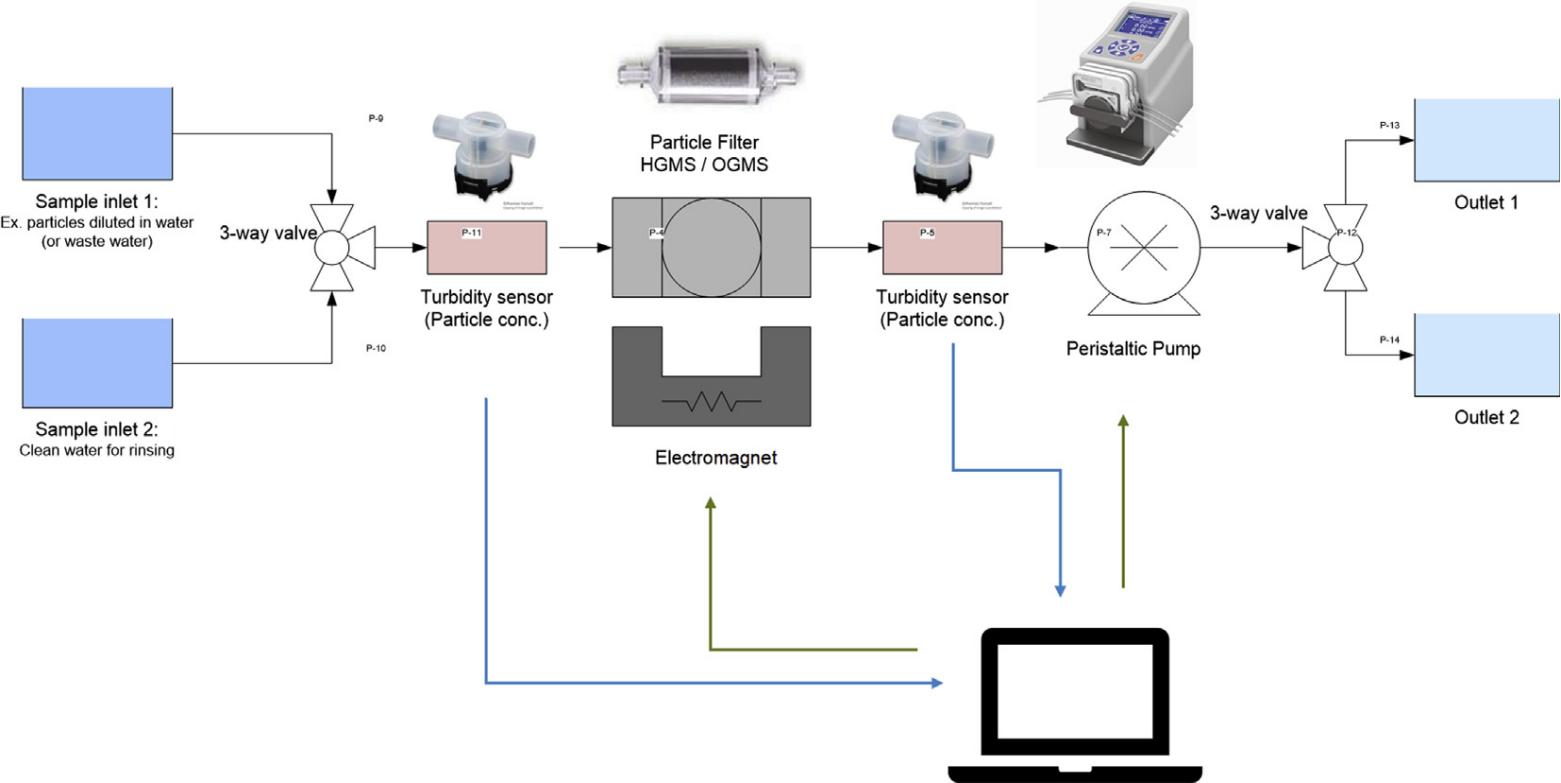
Schematic layout of the separation system used for testing and demonstration purposes. The setup includes turbidity sensors (particle concentration), a peristaltic pump, HGMS filter, an electromagnet and valves, all controlled by a PC.

Several separation concepts have been tested throughout the project to give input to the separation demonstrator (Fig. 1). The simulation indicated quite early on that a HGMS based (high gradient magnetic separator) method should be used rather than an OGMS (open gradient magnetic separator).

The production, application and reuse of magnetic particles was assessed by an attributional life-cycle assessment according to ISO standards (ISO, 2006a, b). The functional unit was defined as 1m^3^ of treated wastewater. The system boundary comprised the complete system of manufacturing of magnetic micro particles and their use for water treatment. Energy and resource use, including raw material production of used chemical intermediate products, were considered for the manufacturing of the particles’ production. The separation process includes energy use and compares different scenarios for the recovery of the micro particles. The data inventory used generic data for the raw materials of particle production. Data for the production of the particles and the separation process were specifically collected from the work in this study. Upstream data to describe the peripheral processes (chemicals, energy and construction) were collected from relevant literature or from life-cycle inventory databases as modules from suitable databases, e.g. Ecoinvent 3.3 (Wernet et al., 2016) and GaBi SP36 (thinkstep AG, 2018).

In this study, only an initial environmental impact assessment was performed, meaning that only the impact on climate change accounting for all direct and indirect emissions of the greenhouse gases carbon dioxide (CO_2_), methane (CH_4_) and nitrous oxide (N_2_O) was included.

## 3 Results and discussion

### 3.1 Experimental acquisition of the data

**Fig. 2:**
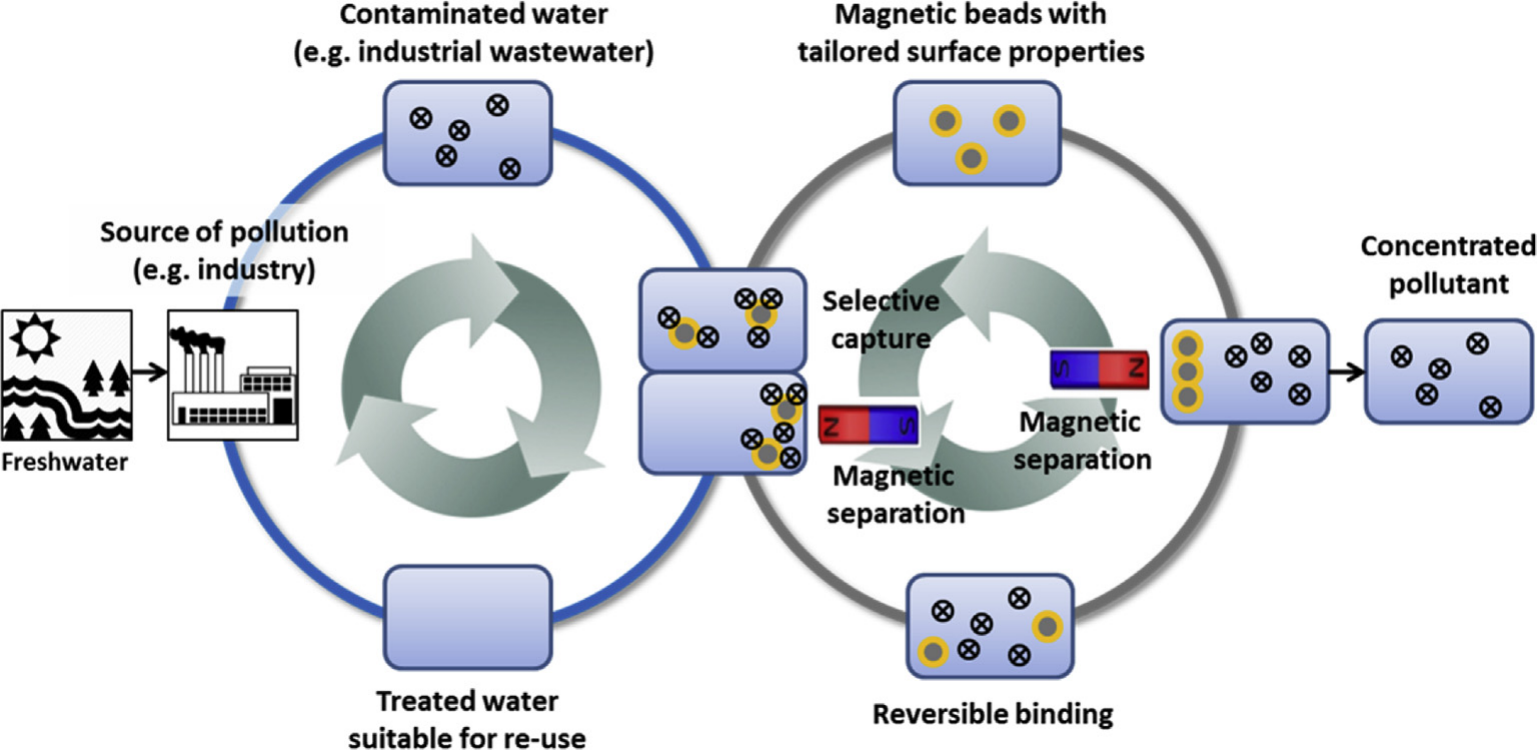
Schematic illustration of the developed and proven concept of functionalized magnetic particles for water treatment.

The project successfully developed a concept showing how functionalized magnetic particles could be utilized for the treatment of water that is contaminated by heavy metals in an industrial setting. Fig. 2 presents a typical situation where the aim is to extract the pollutant with magnetic beads from diluted water, to be further magnetically separated. The collected beads are then entered into a second cycle where they are processed to be recovered.

**Fig. 3:**
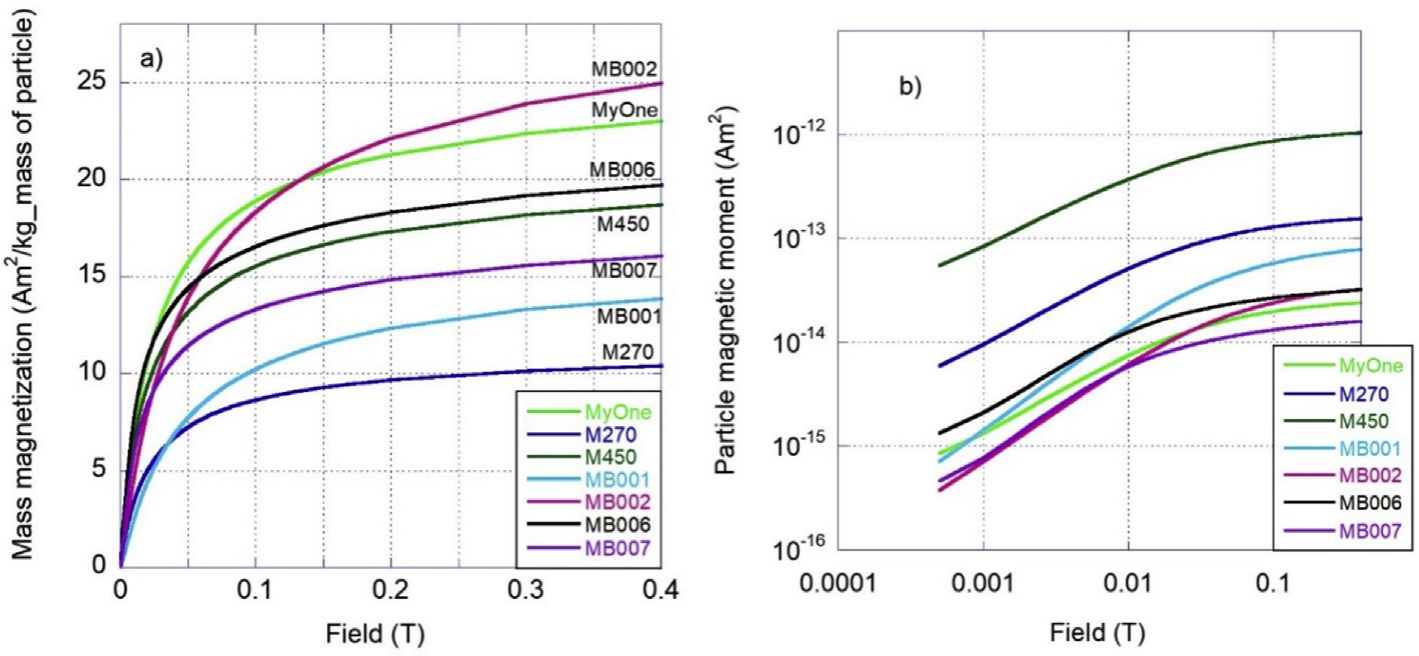
Magnetization versus field at room temperature up to 0.4 T (upper field range for a typical separation magnet), for all particle systems studied during the project. The sample magnetic moment was normalized with the mass of the particles to obtain the mass magnetization in the graph on the left (a) and with the number of particles to obtain the mean value of magnetic moment per particle in the graph on the right (b). The properties of the magnetic particles as indicated in the legends are shown in Table 1. Both the magnetic particles synthesized in this study as well as the commercial magnetic particles (MyOne, M270 and M450 Dynabeads Thermo Fisher Scientific) were used.

To perform the proof of concept for the established model, the different stages including the production of the particles, the functionalization, utilization and separation were set up at lab-scale and tested and evaluated. Iron oxide nanoparticles were prepared and incorporated within the beads. Two different core sizes (7 nm cores and 25 nm cores) were used to synthesize the total magnetic beads with sizes in the 1 μm–2 μm range. The cores are evenly dispersed in the particle matrix. Magnetic properties were assessed. For this, the sample magnetic moment versus field from up to 1 T at room temperature were measured for each particle system and benchmarked against commercial particle systems (M450 with particle diameter 4.5 μm, M270 with particle diameter 2.7 μm and MyOne with particle diameter 1 μm Dynabeads from Thermo Fisher Scientific, Fonnum et al., 2005). The sample magnetic moment was then normalized to the total particle mass to obtain the mean mass magnetization of the particles and to the number of particles to obtain the mean magnetic moment per particle (see Fig. 3).

The magnetic properties at a specific magnetic field strength as shown in Fig. 3 together with the field gradient gives the magnetic force on the particles that moves and separates the particles towards the magnetic separator system. The field and field gradient at the particles are created from the magnetic separator system used. In order to have a large magnetic force on the particles (that gives a large velocity of the particle and therefore a short separation time) the particle magnetic moment shall be as high as possible. It can be observed that the larger sized commercial particles M450 and M270 have a larger particle magnetic moment (Fig. 3) than the other studied particles. This is due to that these particles have a larger size and contain larger magnetic cores in the particles.

**Fig. 4:**
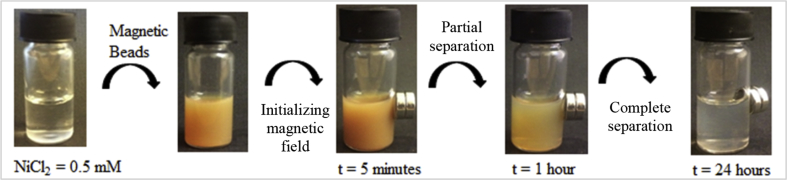
Illustration of the extraction capacity of the functionalized particles.

The aim of using magnetic beads based on magnetic nanoparticles in a polymeric matrix was to take advantage of the preparation route to introduce an ad-hoc surface functionalization in a one-step process. In a nutshell, the process of preparation is referred to as *emulsion solvent evaporation route*, where first an oil-in-water emulsion was prepared using a chelating surfactant for stabilization. The oil pools contained both the magnetic particles and a solvent the polymer was dissolved in. After solvent removal, the polymeric matrix trapped the magnetic cores, while the hydrophobic tails of the surfactants ensured that the chelating headgroups are anchored. In the present case, the dedicated surface-active agents enabled a selective capture and reversible binding of the target pollutants. The surface activity of the surfactants in presence of divalent ions such as Zn^2+^ and Ni^2+^ was also evaluated by surface tension measurements. The extraction capacity of the functionalized particles was then studied by inductive coupled plasma mass spectrometry (ICP-MS). The results showed that the aspartate-based surfactant was the most effective in chelating the divalent species and by taking advantage of the magnetic responsiveness of the particles, extraction levels of >90 % were reached. Fig. 4 shows typical application tests of the particles. Interestingly the presence of NiCl_2_ induced partial aggregation of the beads, which resulted in an easier extraction, as the magnetic response is somewhat proportional to the size of the complexes.

**Fig. 5:**
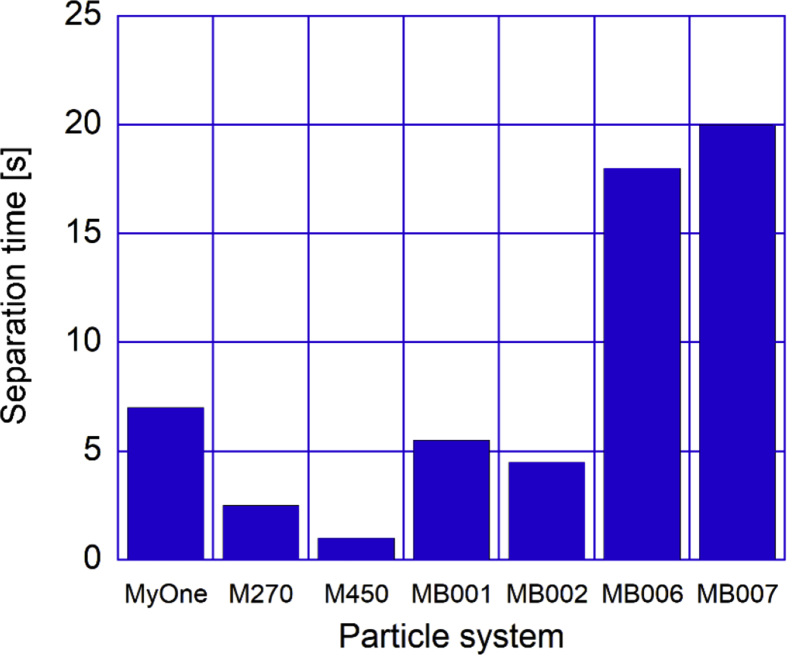
Measured separation time**s** for all particle systems studied in the project, MyOne (1 μm diameter), M270 (2.7 μm) and M450 (4.5 μm) are commercial systems from ThermoFisher and MB01-MB07 (1–2 μm) are produced in the project. The separation time in the optical method was defined as the time when half of the light intensity value as compared to the initial value.

To measure the separation process (separation time), we used an optical method that measured the change in particle concentration in a sample volume by monitoring changes in light transmittance over time (Schaller et al., 2008) as described in the Methods section. The optical setup was used to measure the separation time for all particle systems. In Fig. 5, the separation time results are compiled for the studied particles. Based on these results we can conclude that the MB-particles performs quite well compared to the commercial systems. The fact that the two largest particles M450 (particle size 4.5 μm) and M270 (particle size 2.7 μm) are the fastest to separate shows the importance of the particle size and the magnetic content per particle. MB006 and MB007 are polymer structures covered with small magnetic cores, building up 1.1–1.3 μm large particles. The magnetic content is lower in these particles compared to MB001 and MB002, which probably explains the longer separation time despite the similar sizes.

**Fig. 6:**
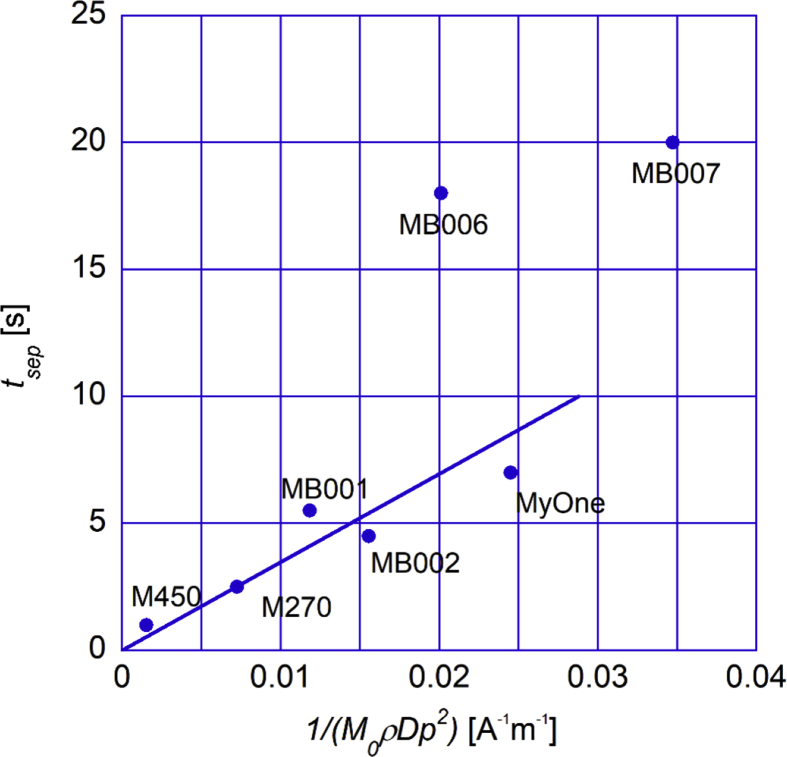
Separation time (in seconds) versus the parameter 1/(*M*_0_*ρD*_*p*_^2^) for the investigated particles indicated in the figure. *M*_*0*_ is obtained from the magnetization measurement results given in Fig. 3a.

An approximate model on how the separation time (*t*_*sep*_) varies with particle magnetic properties, such as the mass saturation magnetization *M*_0_, the diameter of the magnetic particles, *D*_*p*_ and the particle density, *ρ*, is that, *t*_*sep*_ shall linearly scale with 1/(*M*_0_*ρD*_*p*_^2^) (Fonnum et al., 2005). Below in Fig. 6, we have plotted this for the investigated particle systems.

As can be seen in Fig. 6, all investigated particles fall on the same line except MB006 and MB007. The reasons for this can be: 1) these particles are not coherently separated (in the separation process through movements of particle clusters), 2) that the sizes of the cores in these particles are close to the thermal blocking size (Krishnan, 2010), or 3) the magnetic cores are distributed at the particle surfaces.

**Fig. 7:**
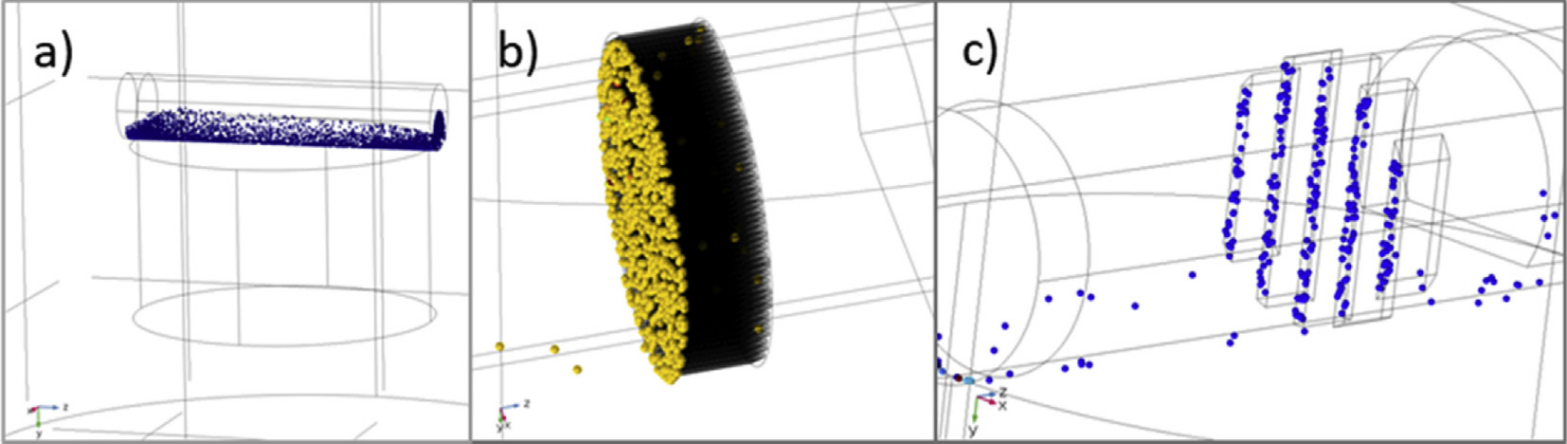
Several FEM-models have been implemented in COMSOL to study different separation concepts including HGMS and OGMS setups. To the left (a) an OGMS system is shown where a flow channel is located on top of a permanent magnet. The particles are typically gathered on the bottom of the channel and the efficiency is given by the strength of the magnet and the flow speed of the fluid containing the particles. The middle figure (b) shows a HGMS setup with a perforated soft magnetic disc inside the flow channel. The disc is magnetized by a permanent magnet beneath the channel. The efficiency of this system is very good due to high gradients and most of the particles are collected in the filter even at higher flow rates. To the right (c) another simulated HGMS filter, built up with vertical soft magnetic bars, is shown.

The experimental study was complemented with FEM modelling (in COMSOL) in order to simulate the separation process (see Fig. 7). The results showed that the model had certain limitations, and it consistently overestimated the separation time as compared to the measurements (optical method). The main reason for this was determined to be the complexity of the physical problem to be modelled including factors affecting the separation process (e.g. particle cooperation effects/particle chain building) that is not considered in the FEM analysis. However, the models provided a good qualitative understanding and helped for the optimization of the separation system (see Fig. 6), for instance how the magnetic separator system shall be designed to optimize magnetic forces on the particles. The simulation indicated quite early on that we should go for a HGMS based (high gradient magnetic separator) method rather than an OGMS (open gradient magnetic separator).

### 3.2 Determination of the key parameters in magnetic depollution based on environmental impact assessment

A life cycle assessment (LCA) investigates the environmental impact of a studied system. The analysis is based on the main function of a given system, defined as the treatment, collection and release of valuable substances or contaminants. To assess environmental impacts in this study, the function of the system was quantified to one thousand liters (1m^3^) of wastewater from industrial processes. By analyzing the different processes, flows and parts of the system, it was possible to identify so-called ‘hotspots’ of environmental concern. Environmental hotspots point out processes that contribute significantly to the total environmental impact and hereby facilitate efforts to minimize the environmental impact of the whole system. The results for the study are divided into two parts: analysis of the hotspots for the manufacturing of particles and their use in the separation and results for different scenarios for the recovery of the particles (S1–S4).

**Fig. 8:**
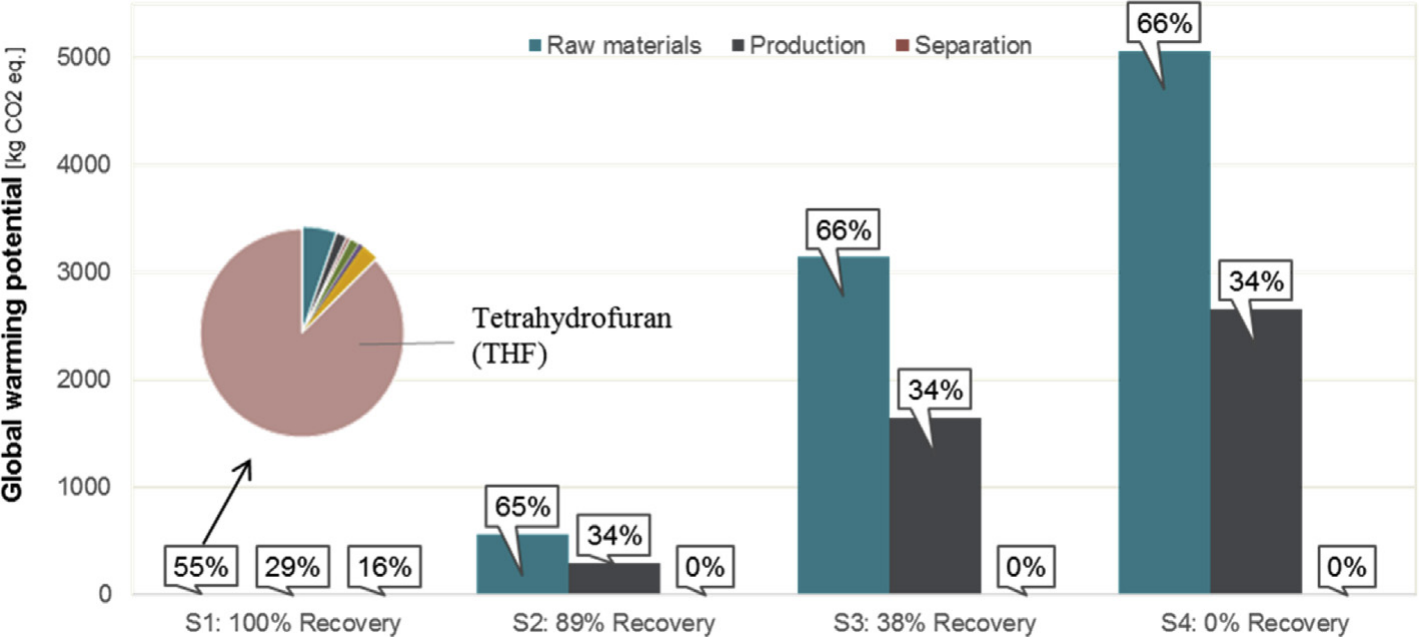
Distribution of impact on climate change for selected scenarios of the system and processes included.

The hotspots analysis for the manufacturing of particles and their use examined the contribution of the included processes and materials on the total impact on climate change. Fig. 8 shows the distribution of the results for the impact on climate change of the different processes in the system.

For the first scenario (S1), where it is was assumed that all particles can be recovered and re-used after the separation process, the production of the raw materials for the production of the particles accounted for more than half of the impact. Within raw material production, one specific raw material (Tetrahydrofuran (THF)) showed a significant impact of more than 85 % of the impact from all raw materials combined. The impact of the production process of the particles contributes second most with 29 %, followed by the separation process, which accounts for 16 % of the total impact on climate change.

To analyze how the recovery rate of the particles affects the total environmental impact of the system, three additional recovery scenarios were examined. Besides the analysis of full recovery (Scenario 1) and no recovery at all (Scenario 4), a specific case scenario (Scenario 2) assumed that 89 % of the particles can be re-used after their first application and a case scenario (Scenario 3) estimated that 38 % of the particles can be re-used. The results for the different scenarios for the recovery rate of the magnetic micro particles are illustrated in Fig. 8. It can be concluded that the total impact on climate change of the studied system is highly sensitive to the recovery rate of the particles. The impact on climate change for systems with full and no recovery of particles differs by more than 835-times. The system for lower recovery rate has a five-fold higher impact on climate change than the system for scenario 2. The impact of the system for scenario 2 is 92-times higher than the system with a full recovery rate of the particles. The contribution of the separation process to the total impact is generally low but decreases for lower recovery rates.

A comparison of the effect of a change in the recovery rate and energy use showed that improvements in the recovery rate have a higher effect on the total environmental impact than a reduced energy use. Furthermore, the results illustrate that further improvements have significantly higher relative potential at higher recovery rates.

## 4 Conclusion

The concept of a novel water treatment method using functionalized μm-size magnetic particles to enable a better and more selective removal of pollutants was successfully demonstrated, by developing tailor-made magnetic particles and implementing at lab-scale, to separate heavy metal ions (Zinc, Nickel) from aqueous solutions.

The magnetic particles produced in the project worked well for magnetic separation, even if additional optimizations are possible. The possibility to recover a major part of the particles in a future up-scaled system looks promising.

The Life Cycle Assessment (LCA) showed that the environmental impact of the system is highly dependent on the recovery rate of the magnetic particles. The absolute impact on climate change varies significantly among the studied scenarios and the different recovery rates. Within the system studied, certain raw materials and production processes for the particles were identified as major contributors to the total environmental impact. The infrastructure required for the manufacturing (e.g. lab equipment) and separation (e.g. magnets) was not included in the analysis. In this study, only an initial environmental impact assessment was performed. That is, only the impact on climate change using the impact category ‘Global Warming Potential (GWP)’ with the unit kg CO_2eq._ was chosen.

The results of this study provide an indication that even if the proof of concepts have been successfully performed, several challenges remain to be solved before a wide implementation of this novel treatment approach can be achieved. These challenges are summarized below and are the target of ongoing work by the authors:

⁃ Enhanced recovery of functionalized particles from realistic water matrices to reduce costs and environmental impacts
⁃ Replacement of certain raw material for the bead production to reduce the overall environmental impact. This may also require an adaption of the production processes.
⁃ Upscaling of the process is needed to identify other limitations. This includes optimizing the magnetic properties of the particles for a resource efficient separation process. The magnetic force on a particle and thus the separation efficiency is dependent on both the applied magnetic field (field strength and gradient) and the magnetic response of the particle.

In conclusion, the water treatment concept presented in this paper provides a novel approach that facilitates a number of possible technological developments and application fields. A successful further development and optimization of the system may not only provide a flexible and tailor-made system for the removal of specific pollutants but also for the recovery of specific resources from various wastewaters.

## Declarations

### Author contribution statement

C. Baresel, V. Schaller, C. Johansson, R. Bordes, V. Chauhan, A. Sugunan, J. Sommertune, S. Welling: Conceived and designed the experiments; Performed the experiments; Analyzed and interpreted the data; Contributed reagents, materials, analysis tools or data; Wrote the paper.

### Funding statement

This work was supported by VINNOVA, the Swedish Governmental Agency for Innovation, within the call Innovationer för ett hållbart samhälle: miljö och transport.

### Competing interest statement

The authors declare no conflict of interest.

### Additional information

No additional information is available for this paper.
